# Nursing Students’ Perception of Nursing as a Career, Outcome Expectations, Job Satisfaction and Informal Workplace Learning

**DOI:** 10.3390/nursrep15060213

**Published:** 2025-06-12

**Authors:** Veronika Anselmann, Sebastian Anselmann

**Affiliations:** 1 Institute for Nursing Science, University of Education Schwäbisch Gmünd, 73525 Schwaebisch Gmuend, Germany; 2 Institute for Education, Vocation and Technique, University of Education Schwäbisch Gmünd, 73525 Schwaebisch Gmuend, Germany; sebastian.anselmann@ph-gmuend.de

**Keywords:** career, learning, job satisfaction, education, nursing

## Abstract

**Background/Objectives**: All countries face a shortage of qualified nurses. Based on the social cognitive career theory (SCCT), it is assumed that individual and environmental aspects are interlinked and determinants in career choice and vocational behaviors. This study aims to determine if nursing students differ in their perceptions of nursing as a career. Furthermore, this study wants to determine if the students in a cluster differed in their outcome expectations, job satisfaction, and informal workplace learning. **Methods**: This study employed a mixed-methods design consisting of two phases: the first involving a pre-study with experts (N = 10) and the second comprising a cross-sectional questionnaire survey. The goal of the pre-study was to find relevant characteristics of the nursing profession. In a cross-sectional study with an online questionnaire, 230 nursing students (N = 230) participated. An inclusion criterion was that participants were enrolled in vocational training to become a nurse. In the questionnaire validated scales were used to ask participants about the characteristics of the nursing profession, their perceptions of nursing as a career, outcome expectations, informal workplace learning, and job satisfaction. **Analysis**: Data analysis included descriptive statistics (e.g., percentage distributions), hierarchical cluster analysis, and analysis of variance (ANOVA). **Results**: The LCA results based on Schwarz’s BIC showed a two-cluster solution (Akaike Information Criterion (AIC) 251.984, Bayesian information criterion (BIC) 265.296, and adjusted Bayesian information criterion (aBIC) 252.622). The results of the ANOVA showed significant differences regarding outcome expectations (F = 22.738; <0.001), the perception of nursing as a career (F = 36.231; <0.001), and the engagement in informal workplace learning activities (F = 20.62; <0.001). For job satisfaction, no significant differences were found. **Conclusions**: Nursing vocational education and training is a vital socialization process in which supervisors can arrange a positive learning climate.

## 1. Introduction

Nurses are the biggest group of professionals in the healthcare system. Since the COVID-19 pandemic, nurses’ working conditions have been characterized by stress [1], workforce shortages [2], burnout [3] and low job satisfaction [4]. Such circumstances are a reason for nurses’ increasing intention to leave [5]. Countries worldwide face a shortage of qualified nurses and little interest among young people in nursing as a career. In the United States, there is a significant gap, requiring an estimated 3 million nurses to meet demand. By 2025, the nurse shortage in the USA could exceed 500,000, while Europe is facing around 590,000 unfilled nursing positions by 2020. In Germany, it is projected that at least 280,000 additional nurses will be needed by 2049 to meet the growing demand [6,7]. Furthermore, nursing education programs have experienced high dropout rates. For England dropout rates are reported in a range of 25 to 40%, and countries like Canada, the United States, and Australia show dropout rates varying from 10% to 50%. In Germany, approximately 27% of nursing trainees drop out of their programs prematurely [8,9]. The research has found that one reason for this can be found in the public image of nursing. Van der Cingel and Brouwer [10] argue that nursing is associated with many stereotypes, such as being in a female job in which helping is the dominant task. Other studies have found that nursing is mainly seen as lacking autonomy and influence [11]. Even though nurses have received much attention during the COVID-19 pandemic, their short-time fame as “heroes” [12] has not radically changed their image in society. Public image can influence nurses’ professional identity development [13]. Nurses’ professional identity means nurses’ actions, knowledge, and skills, as well as their values and beliefs [14]. Studies have shown that nurses with different professional identities use different coping strategies to handle stressful situations caused by the pandemic [15]. Especially for the beginning of a career in nursing, studies have shown that nurses’ professional identity as well as their perception of nursing can influence their professional development [16].

According to social cognitive career theory (SCCT) [17], individual and environmental aspects are interlinked and are significant determinants of career choice and vocational behaviors [18]. Learning experiences that arise through interactions can influence an individual’s confidence in performing career-related tasks [18] (p. 2).

Studies on nursing students’ perceptions have focused on their opinion of clinical settings as learning environments [19] or their experiences during the COVID-19 pandemic [20]. Less is known about nursing students’ thoughts about their target job, career interests, and beliefs. Vocational education and training (VET) in nursing includes theoretical and school-based work in a clinical setting. Some organizations also offer simulation training and lab skills training for their students. Studies have shown that clinical training is often stressful for students [21]. Attending VET can be seen as a socializing process, where nursing students take their first steps in a practical nursing setting. This training occurs as a formal process in the classroom by increasing knowledge while observing role models in clinical reality. It allows students to prepare for future practice and increase their knowledge and skills [22].

The aim of this study is twofold: first, to examine whether nursing students in vocational education and training (VET) differ in their perceptions of nursing as a career; and second, to analyze whether distinct student clusters show differences in outcome expectations, job satisfaction, and informal workplace learning.

## 2. Theoretical Background

### 2.1. The Social Cognitive Career Theory of Career Development

Career choice depends on a variety of “personal, situational, and organizational factors” [23] (p. 289). The SCCT explains career development processes by linking social cognitive variables (such as self-efficacy), individual characteristics (such as personality), goal-related behaviors, and job outcomes, such as satisfaction [24]. The SCCT was developed to “help construct useful bridges, to identify major variables that may compose a more comprehensive explanatory system” for explaining vocational interests [17] (p. 259). Following the SCCT, self-efficacy, outcome expectations, and personal goals explain how interest in vocational occupations can arise. Self-efficacy refers to people’s beliefs about their capabilities “to organize and execute courses of action required to attain designated types of performances” [25] (p. 391). Expectations of the outcome are built on the former experiences of an individual. These expectations describe what an individual thinks and what rewards they can perceive by performing certain actions. Goals are commonly understood as intentions to engage in specific actions or to influence future outcomes [26].

Based on Bandura’s [25] assumptions, Lent and Brown [26] (p. 80) described a conceptual framework that attempts to explain central, dynamic processes and mechanism through which (a) career and academic interests develop, (b) career-related choices are forged and enacted, and (c) performance outcomes are achieved. The authors describe three models that explain how vocational interests are developed, career choices are made, and performance is manifested.

The first model of interest development explains how vocational interests are developed and revised before and while engaging in a specific vocational domain. Vocational interests can be understood as individual patterns of preferences and aversions related to career-relevant activities, which develop over time through repeated experiences, observation of others, and social feedback [26]. These processes are not completed in a vocational orientation phase but are repeated throughout life. Furthermore, Lent and Brown [26] assumed that increased engagement in an activity in a specific domain could lead to a more positive or negative experience, resulting in greater interest or restructuring outcome expectations.

The second model of career choice is an extension of the first model and gives insights into how career choices are made. Lent and Brown [26] (p. 94) observed that choices are “static acts”. They are restructured in the process of gaining experience and receiving feedback. In making career choices, interest in a specific career, the actions implemented to attain goals, and feedback through goal attainment or failures are essential.

The third model of performance links goals and attainment. Performance involves accomplishing specific activities and showing a stable behavior set. Performance is influenced by outcome expectations and the goals an individual has set. Although in VET, performance is predetermined by the curricula, the quality of performance may vary as well as the way performance is achieved [26].

Bandura [25] assumed that individuals and situations interact reciprocally. Personal characteristics, such as beliefs or emotions, external environmental factors, and overt behavior, are closely intertwined. Lent, Brown, and Hackett [27] assume two levels of variables that influence vocation-related behavior and attitudes: cognitive characteristics (such as outcome expectations) and environment-related variables (such as job characteristics). Based on these assumptions, the proposed research model included individual characteristics such as the perception of nursing as a career and outcome expectations. Environmental factors, such as job satisfaction, were also included. The model was complemented by variables focusing on behavior, such as informal workplace learning.

### 2.2. Individual Characteristics, Environmental Characteristics, and Behavior in Nursing

The SCCT is a broad theoretical foundation that shows what variables must be considered when researching career assumptions. Furthermore, it shows how the variables are connected. Using SCCT in nursing research often challenges researchers to clearly distinguish assumptions, attitudes, and behavior based on these assumptions [28]. Price [23] (p. 272) argues that “having the opportunities to understand and experience the professional knowledge and skill of nurses has been shown to greatly influence perceptions of nursing, expectations, and the decision to become a nurse”. Based on this, the SCCT was used as a theoretical base and identified nursing-specific variables by which individual characteristics, environmental aspects, and the components of behavior were captured.

Individual characteristics are the beliefs and assumptions about one’s ability based on learning experiences [25]. Previous personal performance accomplishments or modeling can influence individuals to expect positive or negative effects from their behavior [26]. To identify the individual beliefs nursing students, have about their vocational fields and their activities, perception of nursing as a career, and outcome expectations was included. Nurses’ perception of nursing as a career also concerns nurses’ beliefs and assumptions but focuses more on career attributes such as job security, respect, appreciation, autonomy, and financial remuneration [29]. It is closely linked to nursing students’ “orientation to the profession in terms of orientation to caring, nursing expertise, and the student’s own life experiences” [30] (p. 1056). Bandura [25] (p. 231) believes that people act on their beliefs of “what they can do” and on their beliefs “about the effects” of their actions. These are outcome-based expectations, or “personal beliefs about consequences of performing particular behavior” [27] (p. 41). Bandura [25] observed different kinds of outcome expectations “such as the anticipation of physical, social, and self-evaluative outcomes” [17] (p. 84) that can influence behavior. Studies have shown that outcome expectations play an essential role in nursing, for instance, concerning burnout [24] or nurses’ turnover intention [31].

As an influencing factor the study focused on job satisfaction based on the environmental aspects of the nursing job. Studies have shown that job satisfaction is a critical predictor of turnover in nursing [32] and career identification [33]. Job satisfaction can be defined as “a positive and pleasurable emotional reaction generated by an individual’s overall assessment” [33] (p. 2). Hoppock [34] was the first author to describe job satisfaction as psychological and physiological satisfaction with the activities to be accomplished and the environment at work. He pointed out that satisfaction is the “subjective response to the work environment” [33] (p. 2).

In the 21st century, learning at and for work is perceived as one of the primary sources of knowledge [34]. Considering Billett [35], there are several issues to be dealt with regarding workplace learning. These may consist of three aspects: (1) understanding and making explicit the complex and vast knowledge required for professional practice and identifying ways in which this knowledge can best be learned and developed throughout professional life; (2) an analytical explication of processes that support learning at the individual and organizational level; (3) and thirdly, understanding how learning experiences and educational processes might best be aligned or integrated to support professional learning [36].

In the working environment, continuous staff increases are critical [37]. Billett [35] surmises that learning in the workplace consists of a structure dependent on work experiences to control the exertions necessary to perform the job. In this particular circumstance, learning activities are not linear, formal, impromptu, haphazard or unsystematic. Instead, they are regulated by the requirements of the workplace. Thus, the individual and contextual factors that condition workplace learning also function as determinants.

Frequently, informal workplace learning significantly influences human resource development (HRD) in theory and application, as it encourages an organization’s durability and merit. Informal workplace learning was included in the research model to determine the behavioral component. Informal workplace learning has been described as the “development of knowledge, skills, and attitudes necessary for improving quality and progress of work in situations at the workplace” [37] (p. 436). It is not structured or organized by an institution but allows individuals to learn from their experience in accomplishing daily work activities. Studies have shown that in nursing, cooperation, feedback [38], and role-modeling from the leader [38] are essential sources for informal learning at the nurses’ workplace. Furthermore, studies have shown that it can influence clinical performance [39].

## 3. Method

**Figure 1:**
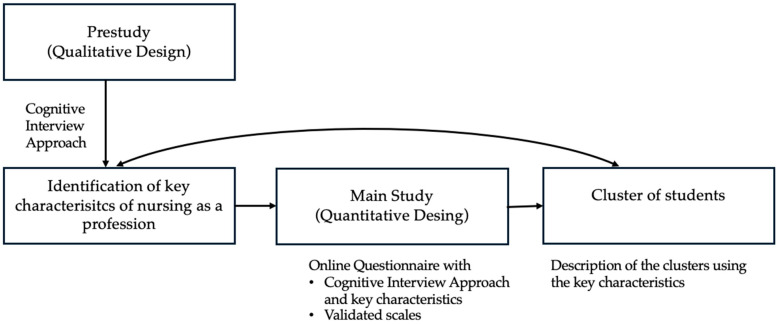
Research design.

This study employed a mixed-methods design, following the framework outlined by Creswell and Plano Clark [40]. In the pre-study, a qualitative approach was used, specifically applying the cognitive interview technique. The goal of this phase was to identify key characteristics of the nursing profession, based on the shared mental model approach. As a result, four distinct characteristics of nursing as a profession were identified. Elements of the cognitive interviewing technique were also integrated into the main study. Participants were asked to choose between the four most frequently mentioned characteristics identified in the pre-study or to suggest an alternative. The main study followed a quantitative design and was conducted using a standardized questionnaire that included several validated scales. The structure and integration of the two phases are illustrated in Figure 1**.**

### 3.1. Pre-Study: Finding Characteristics of Nursing as a Profession

The pre-study was conducted to identify relevant characteristics of the nursing profession. To this end, a preliminary expert study was carried out, following the shared mental model approach [41]. This approach is based on the assumption that individual mental models represent specific knowledge structures. By exploring these models, we aimed to capture characteristics that reflect shared understandings and expectations within the profession. This process helped ensure that the selected questionnaire items are grounded in common professional perceptions.

#### 3.1.1. Sample

All experts (N = 10) who participated in the pre-study had more than 10 years of occupational experience in nursing. They were selected to represent a variety of nursing settings, including geriatrics and acute care. Eight of the participants were female, and two were male. Six were geriatric nurses, and four worked in hospital settings. Experts were purposively recruited based on their extensive professional experience and recognized expertise within their respective fields. Recruitment was conducted through professional networks and contacts at healthcare institutions, ensuring that participants had substantial knowledge and were actively involved in clinical practice or nursing education. This purposive sampling approach was intended to include individuals with a comprehensive understanding of the nursing profession relevant to the study’s aims.

#### 3.1.2. Data Collection

A cognitive interviewing technique was employed as a method to operationalize the shared mental model approach by uncovering individual knowledge structures and areas of conceptual agreement. An open-question mode was used to ensure that participants could describe their own experiences in their own words and highlight their perspectives on nursing [42]. In the questionnaire, experts were asked to complete the following sentence: “When I think of nursing as a profession, I think of…”. They could insert five different characteristics they think in the questionnaire.

#### 3.1.3. Analysis

In line with the shared mental model approach, content analysis serves as a suitable elicitation technique to uncover commonalities in participants’ mental representations. Therefore, summarizing content analysis to identify overlapping themes and shared understandings has been applied. The goal of the qualitative content analysis was to identify commonalities within the data. To achieve this, a semantic analysis was conducted, focusing on the explicit meanings of participants’ responses. According to Kuckartz and Rädiker [43], semantic analysis emphasizes the literal meaning of the text and systematically forms categories based on word meanings. Similar answers were grouped according to their meaning and then inductively assigned to categories. This procedure allowed us to organize the data meaningfully and to uncover shared themes across the expert responses. The content analysis was initially conducted by one researcher, who coded all responses to identify relevant themes and categories. Subsequently, the coding and preliminary findings were thoroughly discussed within the research team to ensure consistency and rigor. The qualitative content analysis was conducted with MAXQDA Version 24.

The resulting category system is presented in Table 1, which provides an overview of the main categories and illustrative examples derived from the data.

### 3.2. The Main Study

For data collection, all vocational colleges with nursing VET in four federal states of Germany were conducted. The principals of the schools were asked to forward the email with the link to the questionnaire to their nursing students. Nursing students were informed that the data collection was anonymous and voluntary.

#### 3.2.1. Method

A cross-sectional study using an online questionnaire with validated scales was conducted. The participants in this study were 230 nursing students (N = 230).

In the online questionnaire, different validated scales were combined. Perception of nursing as a career choice was measured by using an instrument developed by Liaw et al. [28]. An example is “Nursing career provide a chance to achieve higher qualifications”. The instrument was combined with a five-point Likert scale ranging from 1 = agreement to 5 = no agreement. Informal workplace learning was measured with a scale from Decius et al. [44] (e.g., “Before starting a new task, I think about how I can do my work best”). Job satisfaction was measured using a scale from Spector et al. [45]. The scale was combined with a 5-point Likert scale ranging from 1 = agreement to 5 = no agreement (e.g., “I feel satisfied with my chances for salary increases”). Outcome expectations were measured using an instrument from Betz et al. [46]. An example was, “I will be successful in my chosen career”. The Betz et al. [46]. scale was combined with a 5-point Likert scale with responses ranging from 1 = agreement to 5 = no agreement. Finally, a question on the characteristics of the nursing job developed in the pre-study was included.

#### 3.2.2. Ethical Considerations

The ethical committee of the University of Education Schwäbisch Gmünd confirmed that the ethical standards and scientific merit of research were guaranteed. Among the 828 students who accessed the survey, 230 provided complete responses and were therefore included in the final dataset.

#### 3.2.3. Analysis

Cluster analysis aims to identify groups of similar objects based on their characteristics. Hierarchical cluster analysis is particularly suitable when there is no prior hypothesis about the number of clusters. It is well-suited for medium-sized samples and has the advantage of detecting outliers [47]. For the data analysis we used IBM SPSS Statistics 29 and Mplus Version 8. The data analysis included several steps:

1. Factor analysis, descriptive statistical data analysis, and Cronbach’s alpha for all scales
2. Hierarchical cluster analysis using the ward approach [48]. The cluster solution was validated with a latent class analysis (LCA) technique with Mplus using Schwarz’s Bayesian information criterion (BIC) [49].
3. Analysis of the means and standard deviations of the variables for both clusters.
4. Analysis of variance (ANOVA) with Scheffe post hoc test to compare the clusters.

## 4. Results

### 4.1. Results of the Pre-Study

Most participants gave five answers, and only two named four characteristics. A total of 48 characteristics were analyzed with qualitative content analysis [50]. Results of the analysis showed that most of the characteristics could be subsumed in the categories of “empathy”, “competence”, “high workload”, and “joy”. One expert named “helping humans, hard work, time pressure, and being in relation with humans” the five most essential characteristics. Other experts described “feelings of joy, competence, respect, empathy, and nursing crisis”.

#### Integration of Pre-Study Findings into the Main Study

To ensure integration of the qualitative and quantitative components, findings from the pre-study directly informed the design and interpretation of the main study. Specifically, the four key characteristics of nursing as a profession identified in the pre-study were incorporated into the main study questionnaire. Participants were asked to complete the sentence, “When I think of nursing as a profession, I think of …” by choosing one of the four characteristics, empathy, competence, high workload, or joy, or by adding an alternative characteristic. This question combined a closed-ended format with an open-ended option, allowing participants to select from the most frequently mentioned expert-derived characteristics or to suggest new ones. The responses from the main study participants were subsequently used to help characterize the identified clusters. This approach demonstrates a meaningful connection between the qualitative and quantitative phases, ensuring that the findings are integrated rather than presented in isolation throughout the results section.

### 4.2. Results of the Main Study

#### 4.2.1. Sample Characteristics

In our study 230 participants (N = 230) participated. VET of nurses in Germany has been provided by nursing colleges or universities for three years. Colleges and universities are responsible for the theoretical part of the training, whereas hospitals or geriatric homes provide the practical part. Vocational colleges and universities offer nursing training in different settings, such as geriatric care or acute care. Of the participants, 28.9% were nursing students in geriatric nursing, 21.6% were in acute care, 39.2% were in generalist care, and 0.4% attended a study program in nursing at a university. Of the participants, 74% were female, 37.7% were in the first year, 14.6% were in the second year, and 33.2% were in the third year of their training program.

#### 4.2.2. Descriptive Statistics

Cronbach’s alpha for all scales was acceptable, ranging from 0.75 to 0.90. The mean scores and standard deviations of all scales are shown in Table 1. The analysis of the means showed that nursing students had a low level of satisfaction with their jobs.

#### 4.2.3. Cluster Analysis

In the second step, a hierarchical cluster analysis with the ward approach was used by using the Statistical Package for the Social Sciences (IBM SPSS Statistics 29). The results indicated a two-cluster solution for the data. Cluster 1 comprised 135 participants, and Cluster 2 comprised 95 participants. The LCA results based on Schwarz’s BIC also showed a two-cluster solution (AIC 251.984, BIC 265.296, and aBIC 252.622). Table 2 shows the mean and standard deviations for both clusters.

#### 4.2.4. Description of the Clusters

In Cluster 1, 36.4 percent of the nursing students were in a generalist nursing VET program, and 0.8 percent were in a student program; 76.3 percent were female. Participants in Cluster 1 were less satisfied with their jobs. A mean score of 2.55 regarding their perception of nursing as a career indicated that they were not convinced that their job could be a satisfying career.

Most of those in Cluster 1 (42.1 percent) would characterize their future jobs with “empathy”. Of the cluster, 13.7 percent of the nursing students selected “high workload”, 16.8 percent “competence”, and 13.7 percent selected “joy”. Most participants in Cluster 1 gave answers that could be categorized as empathy. They explained it as “a strong feeling of humanity and empathy”. Some also focused on competence as an essential factor in their job, but in combination with “a lot of social and interpersonal contacts”.

In total, 43.2 percent of participants in Cluster 2 were in the generalist VET, and 4.2 percent are students in a study program at a university; 74.4 percent were female. Participants in this cluster were engaged in informal workplace learning activities: 37.9 percent of the participants described their job with “empathy”, 18.9 percent with competence, 27.3 percent with “high workload”, and 9.8 percent with “joy”. Participants in this cluster described their workloads as “a lot of intolerable physical and psychological stress”. Others characterized their jobs as ones in which “interest and competence are necessary”. Empathy and “having fun working with humans” were also described by a participant in this cluster. Table 3 shows mean and standard deviation for each cluster. 

#### 4.2.5. Results of the ANOVA

The results of the ANOVA showed significant differences regarding outcome expectations (F = 22.738; <0.001), the perception of nursing as a career (F = 36.231; <0.001), and the engagement in informal workplace learning activities (F = 20.62; <0.001). For job satisfaction, no significant differences were found.

## 5. Discussion

The results indicate that nursing students differ regarding their perceptions of nursing as a career. The results of this study showed that there are two clusters of nursing students. Nursing students in Cluster 1 were less convinced that nursing could be a satisfying career. They were less engaged in informal learning activities and have lower outcome expectations. When they think of their future profession, they mostly think about empathy and work with humans. Nursing students in Cluster 2 were more engaged in informal workplace learning and had a significant positive perception of nursing as a career. When they thought about their careers, they thought about empathy and competence. Vocational interests and career-related activities could change and be restructured throughout one’s lifespan. They are influenced by individual and environmental aspects that offer learning opportunities and, by this, change in behavior. This study used the SCCT to combine nursing-specific variables and determine how nursing students think about their future jobs. These results indicate that their students can be distinguished into two groups.

The results are consistent with research on nursing students’ motivation and job satisfaction. Messineo et al. [51] found that nursing students differed from low- to highly motivated students. Other studies showed that nursing students’ motivation and interest vary concerning specific settings, such as a low motivation to work in gerontological settings [52]. Saeedi and Parvizy [53] found that increasing nursing students’ academic motivation is necessary. These studies recommended promoting effective strategies to foster and support students’ motivation. Lommi et al. [54] suggested a more targeted recruitment, because in their study they found out that nursing students’ career decisions are based on social models and family advice. According to Ali et al. [55], the desire to engage in socially meaningful work is a significant motivator for entering the nursing profession.

Additionally, understanding the differences in perceptions and motivations among nursing students can help inform curriculum design and teaching strategies. By recognizing the distinct needs and attitudes of different student groups, nursing programs can tailor interventions and educational approaches to foster greater engagement and satisfaction. In particular, addressing the barriers that Cluster 1 students face, such as low engagement and outcome expectations, can improve their overall motivation and commitment to the profession. Furthermore, it is essential for educators and policymakers to consider these findings when creating supportive environments that nurture both career satisfaction and academic success, ultimately leading to a more committed and well-prepared nursing workforce.

The limitation of this study was the small sample size. A larger, more diverse sample could help to better understand the variations in nursing students’ attitudes across different regions and educational systems. It is also important to note that nursing education differs widely across countries. For example, some countries follow a more academic route, where nursing is typically studied at universities, while others offer vocational training or a combination of both. These differences in training pathways may influence how nursing students perceive their future profession and their level of engagement with learning activities.

Additionally, several methodological constraints should be considered. The representativeness of the sample is one such limitation, as the findings may not fully reflect the experiences and perceptions of all nursing students. Furthermore, future research could benefit from incorporating qualitative analyses to provide a deeper understanding of nursing students’ attitudes and experiences.

Moreover, incorporating longitudinal designs could shed light on how perceptions and motivations evolve over time, especially as students progress through their training and encounter real-world experiences. This would offer a deeper understanding of how early career perceptions are influenced by both education and practical exposure to the nursing field.

## 6. Conclusions

In conclusion, this study successfully integrates findings from both the qualitative pre-study and the quantitative main study, offering a comprehensive understanding of the key characteristics of the nursing profession. The qualitative phase identified core themes that informed the development of the quantitative survey, ensuring that the main study was grounded in expert insights. The quantitative results not only validated these themes but also extended them by capturing their prevalence and significance among a broader participant group. This integrated approach highlights the value of combining qualitative and quantitative methods to deepen our understanding and provide more robust evidence for nursing practice and education. The results of this study indicate that nursing students in vocational education and training (VET) can be grouped into distinct clusters based on their perceptions of the nursing profession. These clusters reflect different orientations toward empathy, competence, stress, and job satisfaction. A key conclusion is that VET plays a critical role in shaping and reinforcing these professional perceptions. Given that VET functions not only as a training model but also as a process of professional socialization, it can influence the development of values, attitudes, and competencies that are considered important within the profession. Depending on the structure and quality of the educational experience—especially clinical learning environments—students may adopt differing views of nursing as a career. These findings suggest that the educational context contributes to the formation and differentiation of professional identities during training.

While our study did not directly investigate mechanisms of educational influence, the findings provide a starting point for further exploration. Future studies should examine how different educational systems—such as university-based programs, dual models, or purely vocational training—affect students’ perceptions and career motivation. Comparative research could explore how theoretical emphasis, clinical exposure, and mentorship contribute to these perceptions.

Based on our findings and the existing literature, we propose three areas of improvement for VET programs:

- VET should provide broad and structured opportunities for clinical learning. These experiences must be shielded from excessive stress and workload to ensure students can focus on skill acquisition and reflective practice.
- Clinical supervisors and mentors require ongoing training to enhance their ability to facilitate learning. They should serve as a bridge between practice and theory, helping students connect school-based knowledge with real-world application.
- Coordination between schools and clinical sites must be improved. Educators and supervisors should collaborate closely to align curricular goals with practice environments [56].

Education policy should support these changes through structural reforms—such as clearer definitions of collaborative responsibilities and investments in mentor training. Curriculum design should also focus more strongly on the integration of complex clinical tasks and professional competencies [57].

Our findings resonate with broader discussions about the public image of nursing. A more accurate portrayal of nurses as skilled professionals—rather than relying on emotionally charged metaphors like “angels” or “heroes”—may help attract students who identify with the cognitive and technical demands of the profession [58]. This shift supports the ongoing global professionalization of nursing [59].

In line with Roth et al. [60], addressing push and pull factors in nursing careers, including working conditions and career development opportunities, will be key to ensuring that young people perceive nursing not just as a calling, but as a sustainable and respected profession.

## Figures and Tables

**Table 1:** Category system.

Category	Definition	Example
Empathy	The ability to understand, feel, and respond to the emotions and experiences of patients, demonstrating compassion and emotional support in care.	“Pleasant interaction with clients”
Competence	The possession and application of the necessary knowledge, skills, and professional judgment required to provide safe and effective nursing care	“Good nursing knowledge and competence”
High workload	A demanding work environment characterized by time pressure, staff shortages, and a high number of tasks, often leading to physical and emotional strain	“Nursing is demanding”.
Joy	A sense of personal fulfillment, satisfaction, or emotional reward experienced through meaningful interactions and a sense of purpose in nursing practice	“Enjoyment of work”

**Table 2:** Means, standard deviation, and Cronbach’s’ alpha.

	Mean	Standard Deviation	Cronbach’s Alpha
Perception of nursing as a career 5-point Likert scale, 1 = agreement, 5 = no agreement	2.37	0.58	0.82
Outcome expectations 5-point Likert scale, 1 = agreement, 5 = no agreement	2.20	0.66	0.80
Informal workplace learning 5-point Likert scale, 1 = agreement, 5 = no agreement	2.01	0.55	0.90
Job satisfaction 5-point Likert scale, 1 = agreement, 5 = no agreement	4.28	0.58	0.75

**Table 3:** Mean and standard deviation for each cluster.

	Cluster 1 (N = 135)		Cluster 2 (N = 95)	
	M	SD	M	SD
Perception of nursing as a career	2.55	0.43	2.12	0.52
Informal workplace learning	2.17	0.44	1.87	0.57
Outcome expectations	2.37	0.64	1.96	0.61
Job satisfaction	4.34	0.55	4.21	0.59

## Data Availability

Data available on request due to privacy/ethical restrictions.

## Public Involvement Statement

No public involvement in any aspect of this research.

## Guidelines and Standards Statement

This manuscript was drafted against STROBE Guidelines; von Elm E, Altman DG, Egger M, Pocock SJ, Gotzsche PC, Vandenbroucke JP. The Strengthening the Reporting of Observational Studies in Epidemiology (STROBE) Statement: guidelines for reporting observational studies for observational research. A complete list of reporting guidelines can be accessed via the equator network: https://www.equator-network.org/.
